# Three-Dimensional Carbon Nitride Nanowire Scaffold for Flexible Supercapacitors

**DOI:** 10.1186/s11671-019-2932-z

**Published:** 2019-03-14

**Authors:** Zhiwei Tang, Xueyu Zhang, Lianfeng Duan, Aimin Wu, Wei Lü

**Affiliations:** 1 School of Chemistry and Life Science, Changchun University of Technology, Changchun, 130012 China; 2 Key Laboratory of Advanced Structural Materials, Ministry of Education and Advanced Institute of Materials Science, Changchun University of Technology, Changchun, 130012 China; 3 Key Laboratory of Materials Modification by Laser, Ion, and Electron Beams (Ministry of Education), Dalian University of Technology, Dalian, 116024 China

**Keywords:** Electrochemical supercapacitor, 3D g-C_3_N_4_, PEDOT:PSS, Flexible device

## Abstract

Herein, a 3D composite electrode supported by g-C_3_N_4_ nanowire framework as scaffold and poly(3,4-ethylenedioxythiophene): poly(4-styrenesulfonate) (PEDOT: PSS) as conducting polymer is reported for flexible solid-state electrochemical capacitors. Compared to pure PEDOT: PSS, the composite electrodes have a greatly increased specific surface and showed good electrochemical performance. A specific capacitance of 202 F g^−1^ is achieved, and 83.5% of initial capacitance maintained after 5000 cycles. The device based on the 3D g-C_3_N_4_/PEDOT: PSS electrode also exhibits good performance in capacitance, flexibility, and cycling stability.

**Electronic supplementary material:**

The online version of this article (10.1186/s11671-019-2932-z) contains supplementary material, which is available to authorized users.

## Background

Wearable energy storage devices, especially flexible supercapacitors, are getting extra attention due to their higher cycling stability and power density [1–4]. As for material systems of supercapacitor electrodes, recent researches mainly focus on three principle types: carbon-based high surface area materials (activated carbon, graphene, carbon fibers, and so on), transition metal oxides (MOs), and conducting polymers (CPs) [5–8]. The storage mechanism of the first type is electrochemical double-layer capacitors (EDLCs) while the others are pseudocapacitors [9–11]. Compared to EDLCs, the pseudocapacitors with Faradaic charge storage mechanism show higher specific capacitance, which become an essential part of high-performance supercapacitors. MOs possess high theoretical capacities. However, low conductivity, toxicity, poor stability, and high cost restrict the application of MOs. In contrast, CPs overcoming these problems are suffering the constraint of relatively low mechanical and cycle ability. What is more, the low specific surface is one of the most disadvantages which impede the application of CPs in flexible energy story device.

So far, each of the materials mentioned above has strengths and weaknesses, and none of them is ideal. In order to enhance the performance of devices, compositing materials and optimizing structure are both effective strategies. As for flexible supercapacitors, the composite of 3D EDLC materials and MO (or CPs) pseudocapacitance materials, which keep high electrochemical performance (capacitance, stability) along with well mechanical performance (flexible, light), becomes one of the most suitable choices [12–14]. Although carbon-based materials acted as EDLC materials get some satisfying results, new candidates with competitive performance, low cost, easy fabrication, and eco-friendly properties are still drawing researchers’ attention.

Graphitic carbon nitride (g-C_3_N_4_), a two-dimensional graphene derivative, has been explored due to its interesting electronic feature, low cost, and high environmental-friendly features [15, 16]. In recent years, the application field of g-C_3_N_4_ is mainly focused on photocatalysis [17–22]. Few investigations on the application of supercapacitor for g-C_3_N_4_ got competitive results. Its energy storage potentials are far from fully developed since the molecular structure advantage is not totally explored. The most commonly used microstructure of g-C_3_N_4_ was a 2D structure, while 3D g-C_3_N_4_ structure was rarely reported [23–27]. On the other hand, (3,4-ethylenedioxythiophene): poly(4-styrenesulfonate) (PEDOT: PSS) as a kind of CP is extensively utilized in ES electrode. PEDOT: PSS has high conductivity and relatively much higher chemical and mechanical stability which are basic requirements for wearable energy storage devices. In order to improve its capacitance, enlarging its active surface area is the most direct and effective strategy.

Herein, a 3D g-C_3_N_4_/PEDOT: PSS composite material has been developed where g-C_3_N_4_ nanowire (GCNW) acts as a 3D skeleton structure supporting PEDOT: PSS. The composite materials achieve a specific capacitance of 202 F g^−1^, meanwhile exhibiting an excellent electrochemical performance in the form of all-solid-state flexible supercapacitor. The as-prepared device possessed excellent flexibility and stability. Moreover, the effect of g-C_3_N_4_ ratio on the structure and electrochemical properties had been studied in detail.

## Methods

### Material

Sodium hydroxide (NaOH) and urea were obtained from Beijing Chemical Corp. PEDOT: PSS solution (1.0 wt.% in H_2_O, high-conductivity grade) was purchased from Sigma-Aldrich Co. None of the above products have been further purified.

### Synthesis of g-C_3_N_4_

This preparation used urea as the precursor. Ten grams of urea was heated to 550 °C (10 °C min^−1^) and kept for 2 h in a muffle furnace, producing the yellow powder.

### Three-Dimensional Fabrication of the GCNW

Briefly, 500 mg CN power was mixed with 20 ml of aqueous NaOH and stirred at 60 °C for 12 h. The sealed flasks were ultrasonic cleaned for 2 h. The suspension was dialyzed to remove the excess NaOH. The final pure g-C_3_N_4_ nanowire aerogel was obtained through freeze-drying.

### Three-Dimensional Preparation of GCNW/PEDOT: PSS Composite Material

The composite materials were prepared with different mass ratios of g-C_3_N_4_ nanowire hydrogels (6 mg ml^−1^) to PEDOT: PSS, namely 10%, 20%, 50%, and 80% GCNW/PEDOT: PSS. The homogeneous solution had been gotten after 12 h of stirring. Finally, the product was obtained using the freeze-drying process. The pure PEDOT: PSS thin film was prepared by filtration method for comparison.

### Characterization

The morphologies and structures of samples were characterized by field emission scanning microscopy (FESEM, 7610, JEOL), transmission electron microscopy (TEM, Tecnai F20), and D-MAX II A X-ray diffractometer (XRD). Fourier transform infrared spectroscopy (FTIR) was carried out using Nicolet-6700 (Thermofisher). X-ray photoelectron spectroscopy (XPS) measurements were tested with ESCALABMK II X-ray photoelectron spectrometer.

### Electrochemical Measurement

Electrochemical performance was carried out using a CHI 660E electrochemical workstation. In the three-electrode configuration, the platinum foil and saturated calomel (SCE) electrodes were used as counter and reference electrodes. The working electrodes were prepared by pressing the composite on a carbon cloth with a loading amount 1 mg cm^−2^. The electrolyte was 1 M H_2_SO_4_. Cyclic voltammetry (CV) and galvanostatic charge/discharge (GCD) curves were tested at the potential range of 0 V to 1 V. The electrochemical impedance spectroscopy (EIS) measurements were recorded under an open circuit potential in the frequency range of 1–10^5^ Hz with a modulating amplitude of 5 mV.

For the two-electrode devices, 2 mg of active material was loaded on the carbon cloth as working electrodes. Then, a small amount of H_2_SO_4_/PVA hydrogel was dripped on the non-woven fabric (NKK-MPF30AC-100) as a separator. Finally, the separator was placed between two working electrodes to assemble a symmetrical capacitor. Electrochemical testing of two electrodes was carried out in a CHI 660E electrochemical workstation.

The specific capacitance of a single electrode (*C*_*m*_) was calculated using the charge integrated from CV curves according to the following formulas:

1 $$ {C}_m=\frac{1}{Uvm}{\int}_{U^{-}}^{U^{+}}i(U)\mathrm{d}U $$

where *U* is the voltage window (*U=U*^*+*^*-U*^*−*^), *m* is the mass of active materials in one electrode, and *ν* is scan rate (mv s^−1^) of the CV curve.

Subsequently, the energy density (*E*) and power density (*P*) of ES were calculated using the following formulas:

2 $$ E=\frac{1}{2}C{U}^2 $$

3 $$ P=\frac{E}{\Delta t} $$

where *C* is the specific capacitance value of the supercapacitor, *U* is the voltage window, and Δ*t* is the discharge time in GCD.

## Results and Discussion

**Fig. 1:**
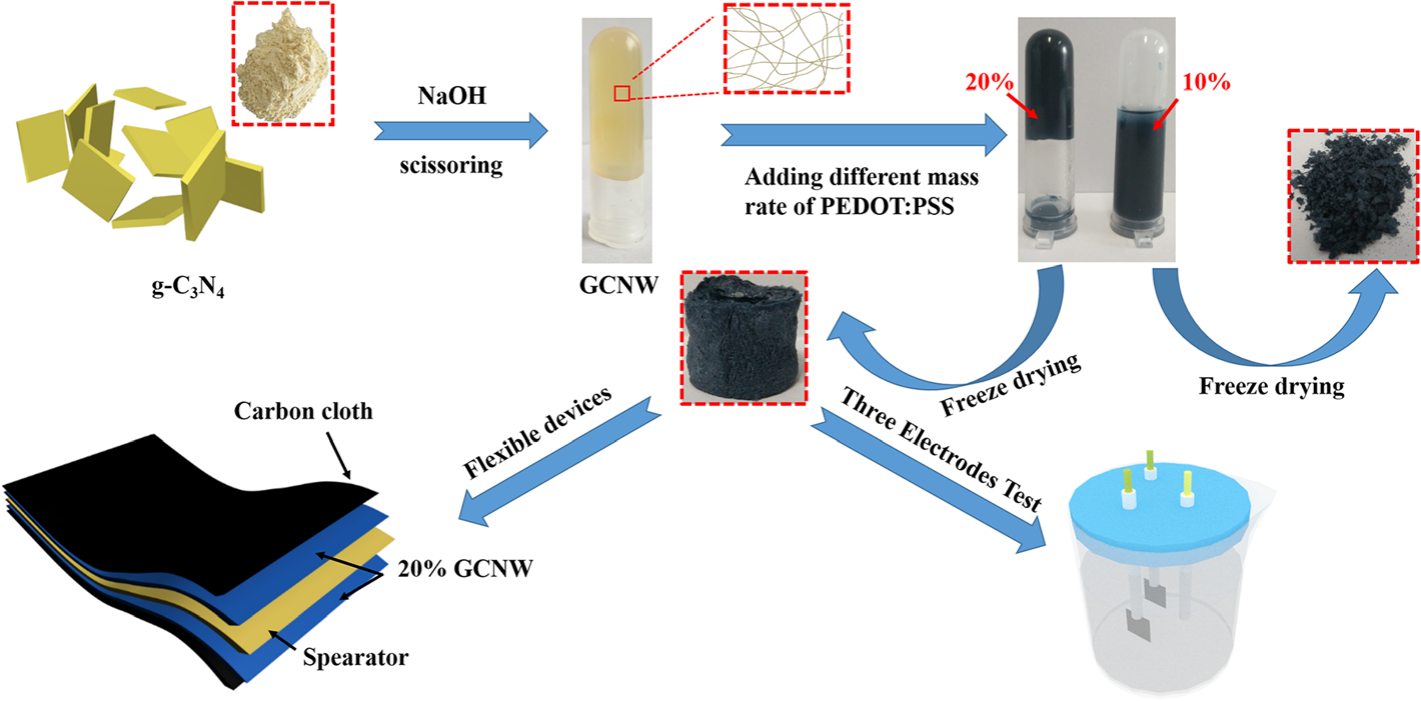
The experimental procedures of GCNW/PEDOT: PSS composite material and flexible device

The experimental procedures and flexible device are shown in Fig. 1. As can be seen, the mass ratio of the composite can affect its structure significantly; the as-prepared composite can hold a well 3D structure when the GCNW mass ratio is not lower than 20%, while the 3D structure would be wrecked as the concentration of PEDOT: PSS was too high (90%). What is more, the concentrations of sodium hydroxide have a substantial influence on the microstructure of g-C_3_N_4_ (Additional file 1: Figures S1–S3). When the concentration of sodium hydroxide is lower than 3 M, the layer structure of g-C_3_N_4_ cannot be cut sufficiently, and no self-supporting 3D structure can be acquired. When the concentration of sodium hydroxide was too high (like 8 M), the GCNW would be cut short, and the 3D structure also collapsed after the freeze-drying process. In this work, 3 M is a suitable concentration for sodium hydroxide treatment due to the well self-supporting 3D structure.

**Fig. 2:**
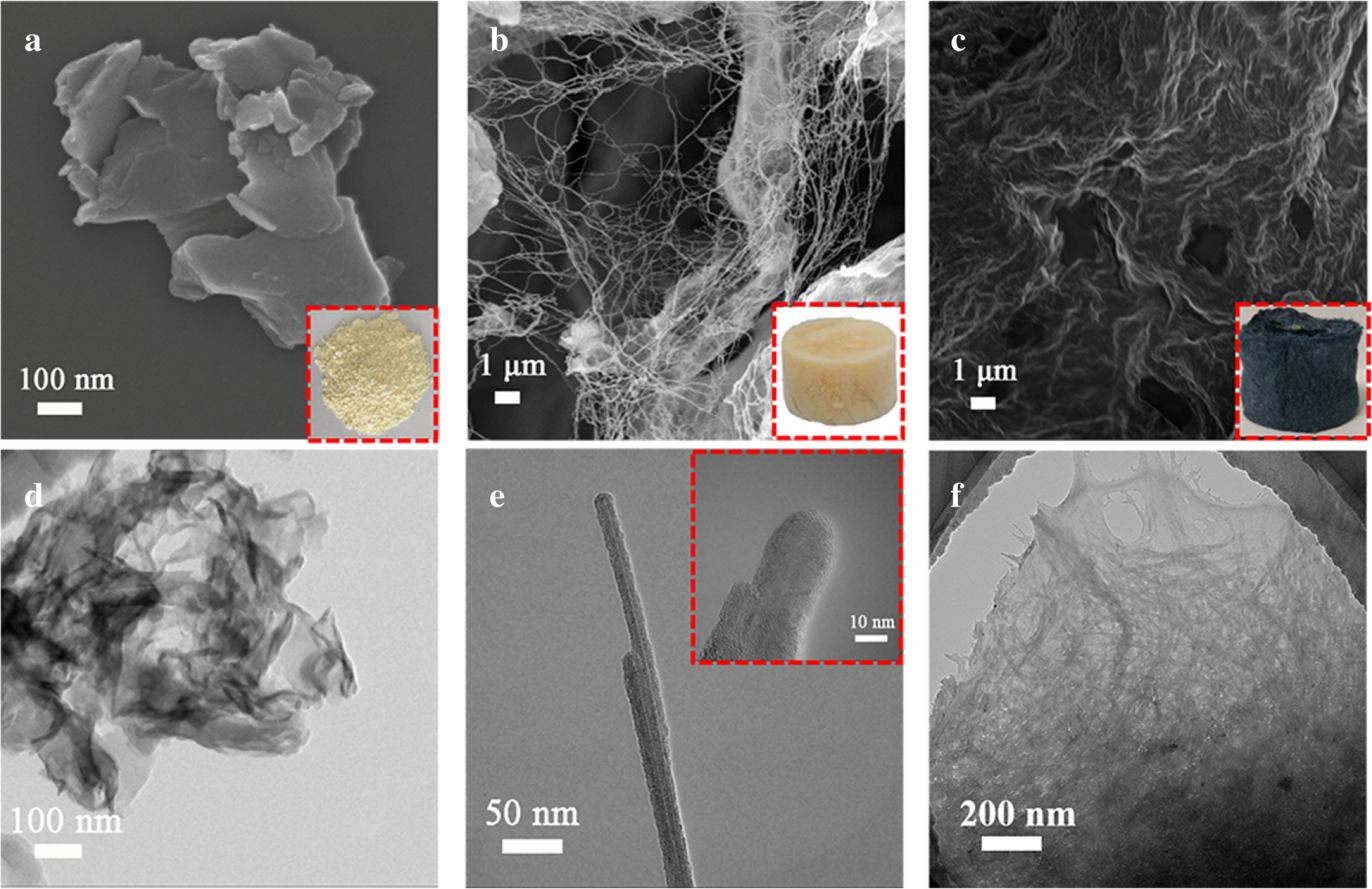
Structure characterization. FESEM images of g-C_3_N_4_ (**a**), GCNW (**b**), and 20% GCNW/PEDOT: PSS (**c**). TEM images of g-C_3_N_4_ (**d**), GCNW (**e**), and 20% GCNW/PEDOT: PSS (**f**) with 3D structure

The scanning electron microscopy (SEM) images of Fig. 2a and b demonstrate the transformation of g-C_3_N_4_ from layer structure to wire structure, and then, the 3D framework has been achieved using the freeze-drying process. Moreover, the 20% GCNW/PEDOT: PSS composite preserves the 3D framework as shown in Fig. 2c. The digital photograph of the sample appears in the corresponding insets. Comparing the transmission electron microscopy (TEM) images of g-C_3_N_4_ and GCNW in Fig. 2d and e, the as-prepared GCNW exhibits 10 nm in width and hundreds of nanometers in length, which is very suitable as a skeleton material. Figure 2f is the as-prepared GCNW after freeze-drying, which indicated a clear 3D structure. The TEM image of 20% GCNW/PEDOT: PSS composite is shown in Additional file 1: Figure S4 where the 3D structure can also be distinguished. The 3D composite structure can increase the electrochemical active sites and narrow the ion transport distances, which would be a benefit for the improvement of capacitance. Based on the Brunauer-Emmett-Teller measurement (BET) results (Additional file 1: Figure S5), the specific surface of GCNW and 20% GCNW/PEDOT: PSS is 82.67 m^2^ g^−1^ and 69.86 m^2^ g^−1^, respectively. It is worthy to mention that the specific surface of pure PEDOT: PSS is extremely low while the as-prepared pure g-C_3_N_4_ nanosheets can reach up to 149.45 m^2^ g^−1^, but both of their capacitances are low. The detail of electrochemical characters will be discussed later.

**Fig. 3:**
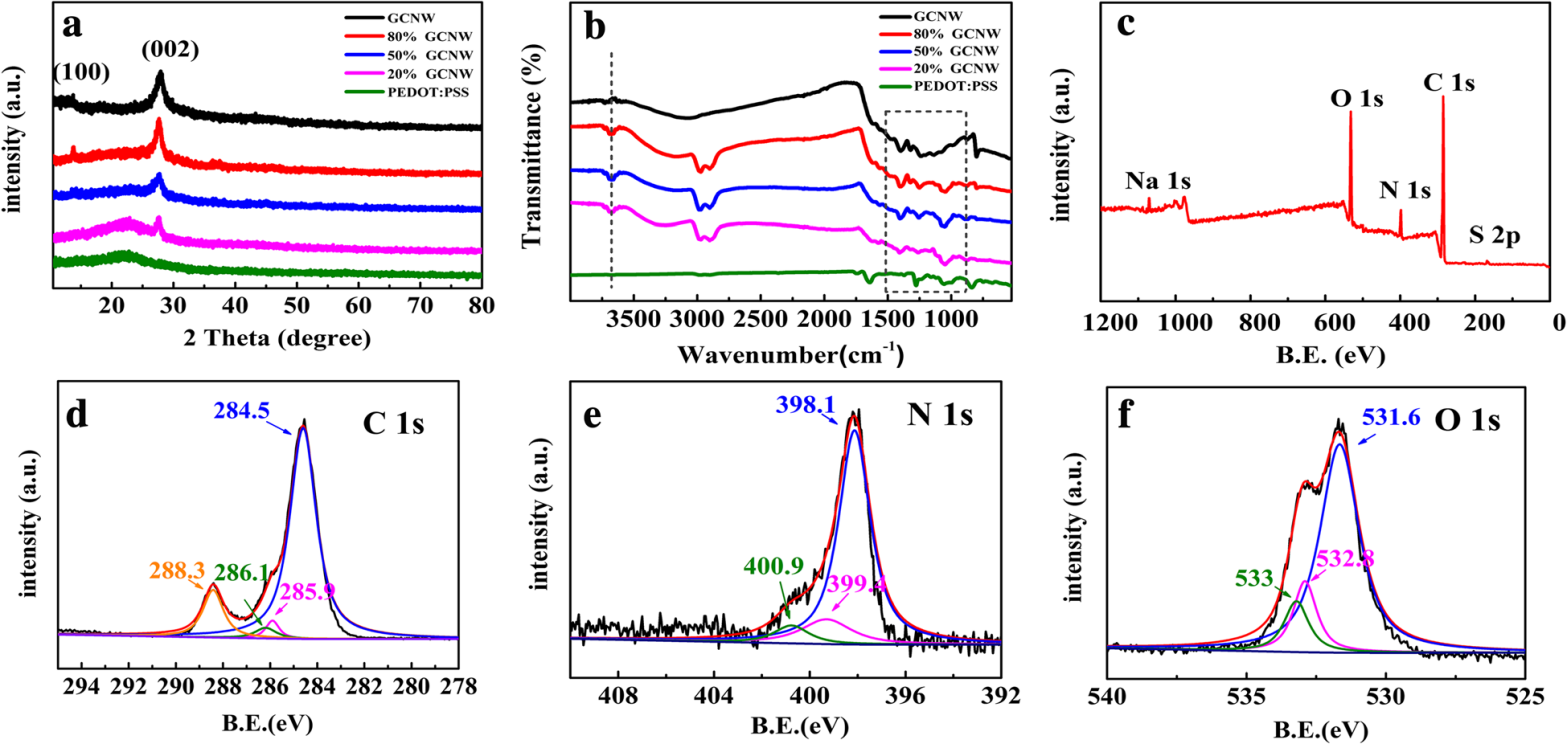
**a** XRD patterns and **b** FT-IR spectra of GCNW, PEDOT: PSS, and GCNW/PEDOT: PSS composite samples with different content ratio. **c** XPS survey spectra of 20% GCNW. The high resolution of C 1 s (**d**), N 1 s (**e**), and O 1 s (**f**) XPS spectra of 20% GCNW

The crystal structures of the sample are shown in Fig. 3a. The GCNW has two clear peaks at 13.84° and 27.81° corresponding to (100) and (200) planes of g-C_3_N_4_, respectively [15]. The broad diffraction peak ranging from 15° to 30° is attributed to PEDOT: PSS [28], and the intensity weakened with the increase of GCNW ratio. The fourier transform infrared spectroscopy (FTIR) spectra were studied to investigate the atomic structure of the as-prepared samples (Fig. 3b). For GCNW, several strong peaks around 804 cm^−1^ are due to tri-s-triazine units and that at 1299, 1350, 1431, 1533, and 1605 cm^−1^ are attributed to C-N heterocycles in GCNW. The peaks between 3000 and 3500 cm^−1^ result from −NH_X_ and −OH vibration modes of GCNW [16, 29]. The resultant FTIR spectrum of pure PEDOT: PSS is well consistent with the previous report [30, 31]. Based on these results, the GCNW/PEDOT: PSS composites are physical mixtures where the GCNW and PEDOT: PSS hold their inherent atom structure, and the bond characters do not change. Figure 3c shows the X-ray photoelectron spectroscopy (XPS) survey spectrum of the GCNW/PEDOT: PSS. The peaks corresponding to C 1s, O 1s, N 1s, S 3p, and Na 1s are observed clearly. The Na 1s peak located at 1047.5 eV comes from the sodium hydroxide which is used to shear g-C_3_N_4_ nanosheets. The C 1s spectrum includes four peaks at 284.5 eV, 285.9 eV, 286.1 eV, and 288.3 eV which correspond to C–C, C–N, C–S, and C=O peaks, respectively (Fig. 3d). Figure 3e is an N 1s spectrum. The peak at 398.1 eV is due to sp^2^ N atoms in C–N=C, and the peaks at 399.4 eV and 400.9 eV are due to N in N–(C)_3_ and C–N–H, respectively. For the O 1s spectra in Fig. 3f, the peaks at the binding energy of 531.6eV, 532.8eV, and 533 eV are observed, which correspond to C=O, C–O and −OH, respectively. The XPS results are consistent with the previous tests and also indicate that the capacitance which was tested later only comes from the g-C_3_N_4_ and PEDOT: PSS.

**Fig. 4:**
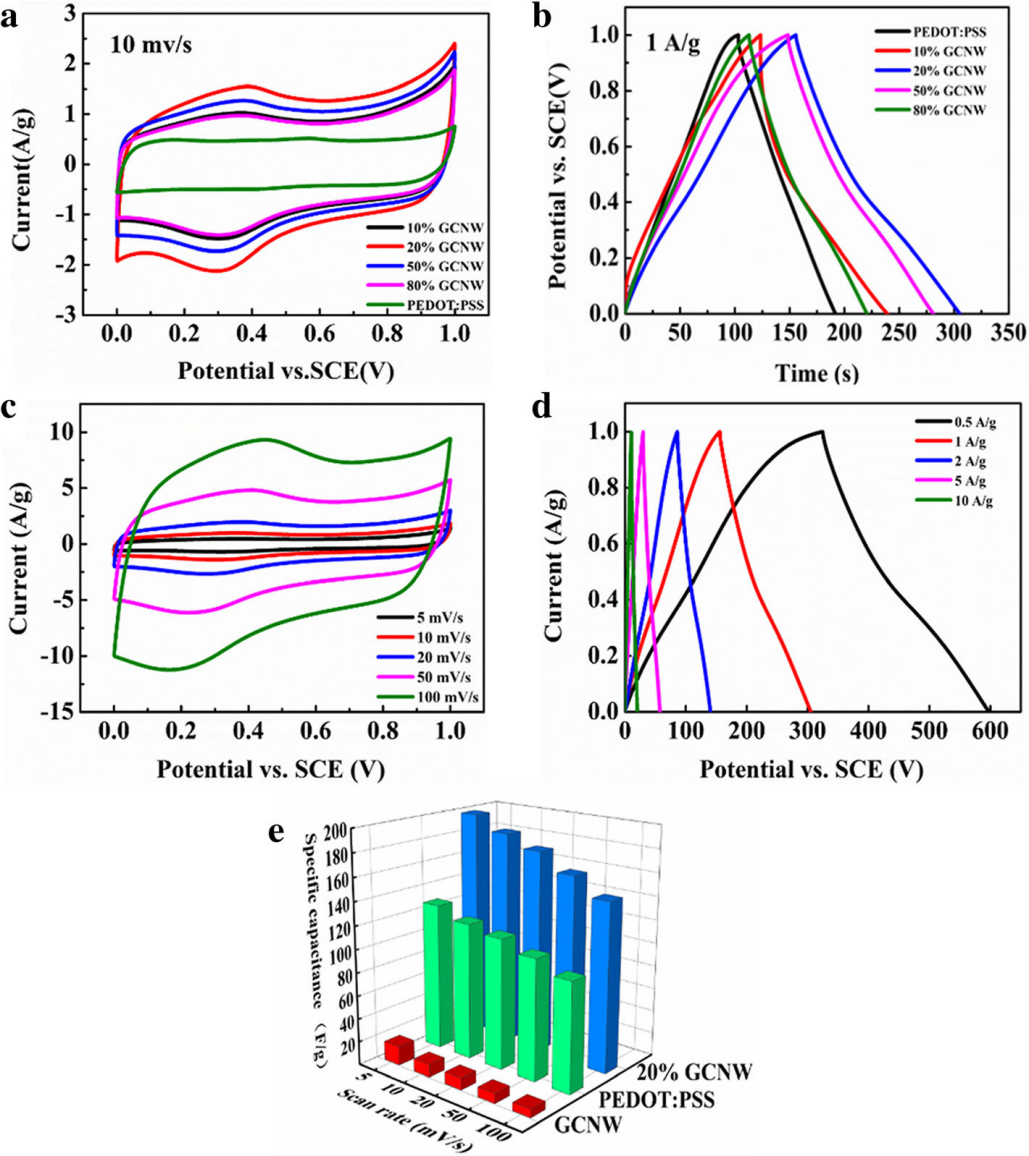
The electrochemical performances of GCNW, PEDOT: PSS, and GCNW/PEDOT: PSS samples with different content ratio of GCNW and PEDOT: PSS. **a** Cyclic voltammograms at the scan rate of 10 mv/s. **b** Galvanostatic discharge curves at current densities of 1 A g^−1^. **c** Cyclic voltammograms with scan rate from 5 mv s^−1^ to 100 mv s^−1^. **d** Galvanostatic discharge curves at various current densities. **e** Specific capacitances of GCNW, PEDOT: PSS, and 20% GCNW/PEDOT: PSS at different scan rate

The performance of the GCNW/PEDOT: PSS as an electrode material for electrochemical was investigated using cyclic voltammetry (CV) measurements and galvanostatic charge/discharge (GCD) through the three-electrode method. Figure 4a exhibits the CV results of the electrodes prepared with different mass ratios. As can be seen, there is no obvious redox peak in all of the results and the electrode of 20% GCNW/PEDOT: PSS gets the biggest integral area which means the maximum capacitance. Meanwhile, these results are certified by the GCD test in which the 20% GCNW/PEDOT: PSS electrode also exhibits the longest time of charge and discharge (Fig. 4b). Figure 4c is the result of the 20% GCNW/PEDOT: PSS measured at different scanning rates. With the increasing scanning rate, the curved profile has no significant change, exhibiting a good rate performance [32–34]. In Fig. 4d, the GCD curves of the 20% GCNW/PEDOT: PSS under different current densities show good symmetry which proves a good electrochemical reversibility [35]. Fig. 4e measures the specific capacitance values of pure GCNW, PEDOT: PSS, and 20% GCNW/PEDOT: PSS composite electrodes. The specific capacitance value of 20% GCNW/PEDOT: PSS is 202 F g^−1^ at 5 mv s^−1^, 46.9% higher than that of pure PEDOT: PSS. To our knowledge, the present 20% GCNW/PEDOT: PSS electrode material is superior to previous reports for C_3_N_4_-based electrodes. In fact, this result is even higher than some carbon-based composite (Additional file 1: Table S1) [36–45]. The improvement should mainly come from the 3D structure to prevent PEDOT: PSS from aggregation providing a higher active surface, which is verified by BET result. Although the specific surface of pure g-C_3_N_4_ is higher than PEDOT: PSS, the capacitance of g-C_3_N_4_ is much lower than that of PEDOT: PSS due to the material nature factor and the storage mechanism. However, the 20% GCNW/PEDOT: PSS electrode gets the maximum capacitance. Therefore, a suitable structure is as important as materials to get excellent performance. In this work, the capacitance of GCNW/PEDOT: PSS electrodes is improved with the GCNW ratio decrease, until it reaches 10% where the 3D structure has been destroyed as shown in Fig. 1.

**Fig. 5:**
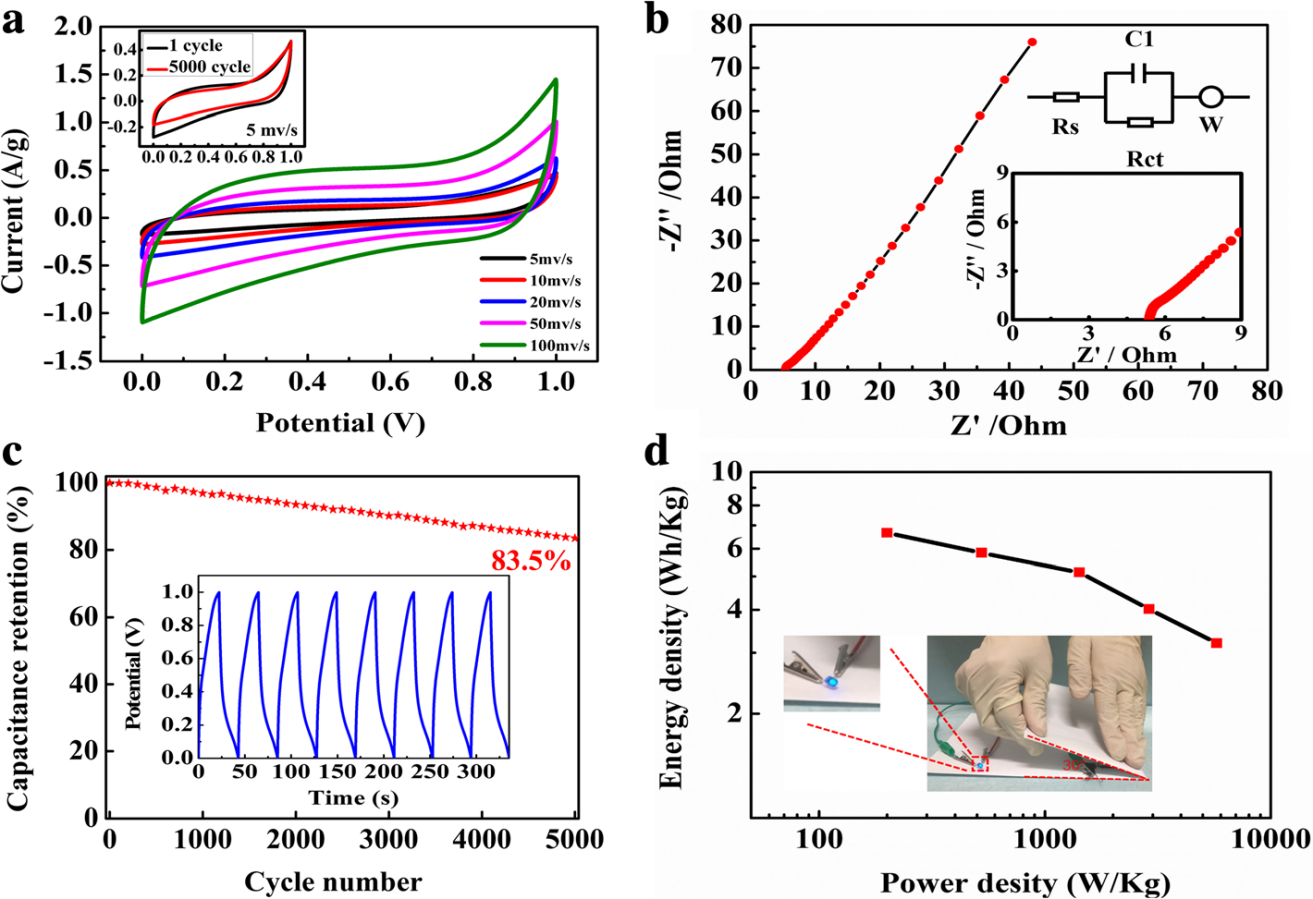
**a** The CV curve of the single device. **b** The EIS of the device. **c** The cycling stability of the device. **d** Power density and energy density of the device

**Fig. 6:**
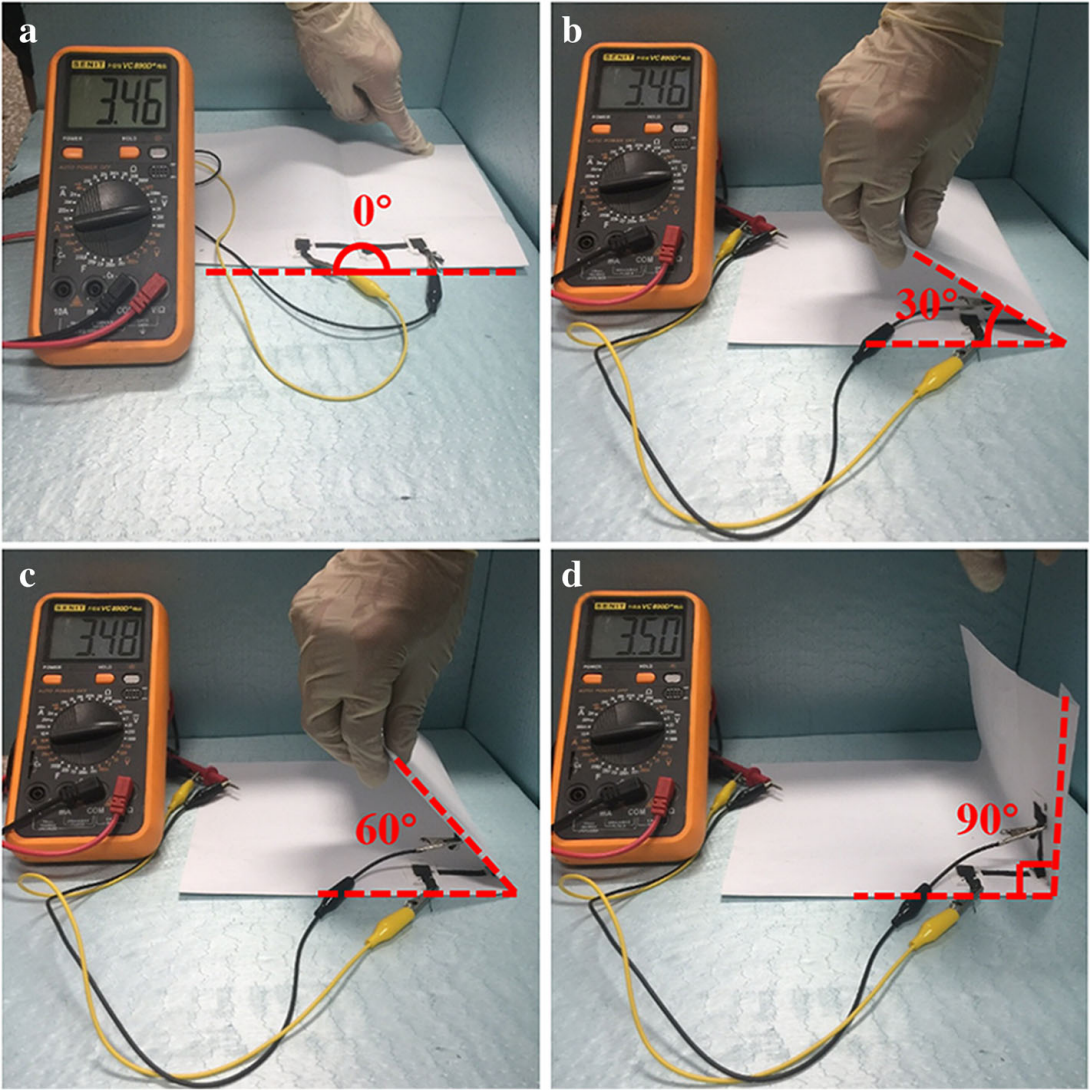
The voltage value of flexible solid-state supercapations based on 20% GCNW under different bending angles (a: 0°, b: 30°, c: 60°, d: 90°)

The symmetric supercapacitors were prepared by assembling 20% GCNW/PEDOT: PSS pressed on a carbon cloth as an electrode (Fig. 1). Figure 5a presents the CV curve of a single device under the voltage window 0–1.0 V with different scan rates. The curve shows a good symmetrical rectangular shape, and the area exhibits a little decrease after 5000 cycles (inset). The specific capacitance is 78 F g^−1^ at the scanning rate of 5 mv s^−1^. Figure 5b is the electrochemical impedance spectroscopy (EIS) of the device. The inset of corresponding figures shows a magnified area of the high-frequency region and the fitting circuit of impedance. The Nyquist impedance plot consisted of straight lines at low frequency and semicircular curve at the high-frequency region. The semicircle in the high-frequency zone is mainly controlled by reaction kinetics, and the low-frequency zone line is controlled by the diffusion of ions. Since C_3_N_4_ is a low conductivity material, the equivalent series resistance (ESR) value of 5.41 Ω is higher than some other works [46–48]. In Fig. 5c, the capacitor maintenance rate is 83.5% after 5000 cycles under the current density of 1 A g^−1^. The loss mainly comes from the PEDOT: PSS component as poor cyclic stability is the fundamental shortcoming of conducting polymers [5–8]. Fig. 6 exhibits the flexible and stable performance of the device. In the digital photo, three devices were connected in series and the discharging voltage was 3.46 V, 3.46 V, 3.48 V, and 3. 50 V with bending angles 0°, 30°, 60°, and 90°, respectively. The flexible device possessed capacitance retentions over 80% after 2000 bending cycles with 90° (Additional file 1: Figure S11). The plot of energy density as a function of power density is shown in Fig. 5d. The energy density of 6.66 Wh Kg^−1^ is achieved at the power density of 200 W Kg^−1^.

## Conclusion

In summary, for the first time, 3D GCNW/PEDOT: PSS composite materials have been prepared and applied as an electrode of flexible supercapacitor successfully. Due to the improvement of the active surface, the capacitance of the composite reached 202 F g^−1^ in the three-electrode system and 78 F g^−1^ in the symmetric device at the scan rate of 5 mV s^−1^, resulting in a high energy density of 6.66 Wh Kg^−1^. The 3D structure was of great significance to enhance electrochemical performance. The as-prepared device also exhibited excellent flexible and stable performance in the bending cycle test. Taking into account the cost and preparation convenience, the results obtained herein open new prospects for 3D g-C_3_N_4_/CP composite as an efficient electrode material in flexible energy storage device and commercial applications.

## Additional file

Additional file 1: **Figure S1.** The digital photo of g-C_3_N_4_ hydrogel treated with different concentrations of sodium hydroxide. From left to right: pure g-C3N4 powder, g-C_3_N_4_ hydrogel treated by 1 M sodium hydroxide, g-C_3_N_4_ hydrogel treated by 3 M sodium hydroxide, g-C_3_N_4_ hydrogel treated by 5 M sodium hydroxide, and g-C_3_N_4_ hydrogel treated by 8 M sodium hydroxide. **Figure S2.** The digital photo of g-C_3_N_4_ aerogel treated with different concentrations of sodium hydroxide corresponding to Figure S1. As can be seen, the g-C_3_N_4_ aerogel treated by 3 M and 5 M sodium hydroxide can hold well 3D structure, while the other two showed power-like structure. **Figure S3.** (a) TEM image of pristine g-C_3_N_4_; TEM image of GCNW after treatment with different concentrations of sodium hydroxide (b: 1 M, c: 3 M, d: 5 M). **Figure S4.** (a) SEM image of PEDOT: PSS. The illustration in the upper right corner is the photograph of PEDOT: PSS. (b, c) TEM images of 20% GCNW. The extracted elemental mapping images of C, N, O, and S, which indicate the homogeneous distribution of g-C_3_N_4_ nanowires and PEDOT: PSS. **Figure S5.** N_2_ sorption isotherms of g-C_3_N_4_ (a), GCNW (b), 50% GCNW/PEDOT: PSS (c), 20% GCNW/PEDOT: PSS (d). **Figure S6.** (a) S2 s XPS spectra of 20% GCNW. (b) Raman spectra of different mixing ratios of GCNW and PEDOT: PSS. In Figure S6b, two strong absorption peaks in the 1434 cm^−1^ and 1515 cm^−1^ region correspond to the symmetry C_α_ = C_β_ (−O) stretching mode and the asymmetric C=C stretching mode which are characteristic of PEDOT: PSS. **Figure S7.** Electrochemical properties of pure PEDOT: PSS: (a) CV curve and (b) GCD curve. **Figure S8.** Electrochemical properties of 10% GCNW: (a) CV curve and (b) GCD curve. **Figure S9.** Electrochemical properties of 50% GCNW: (a) CV curve and (b) GCD curve. **Figure S10.** Electrochemical properties of 80% GCNW: (a) CV curve and (b) GCD curve. **Figure S11.** CV curves of the flexible device after 2000 bending cycles with 90°. **Table S1.** Summary of the capacitive performance of the supercapacitors based on similar structures. (DOCX 6879 kb)

## Abbreviations

BET: Brunauer-Emmett-Teller
CPs: Conducting polymers
CV: Cyclic voltammetry
EDLCs: Electrochemical double-layer capacitors
EIS: Electrochemical impedance spectroscopy
ESR: Equivalent series resistance
FESEM: Field emission scanning electron microscopy
FTIR: Fourier transform infrared spectroscopy
g-C_3_N_4_: Graphitic carbon nitride
GCD: Galavanostatic charge/discharge
GCNW: g-C_3_N_4_ nanowire
MOs: Transition metal oxides
PEDOT: PSS: (3,4-ethylenedioxythiophene): poly(4-styrenesulfonate)
TEM: Transmission electron microscopy
XPS: X-ray photoelectron spectroscopy
XRD: X-ray diffraction patterns
