# Supplementary material for: Three-Dimensional Carbon Nitride Nanowire Scaffold for Flexible Supercapacitors
Source: Nanoscale Res Lett. 2019 Mar 14;14:98. doi: 10.1186/s11671-019-2932-z (PMC6419655; doi:10.1186/s11671-019-2932-z)
Supplement: Supplementary file 1 — Figure S1. The digital photo of g-C3N4 hydrogel treated with different concentrations of sodium hydroxide. From left to right: pure g-C3N4 powder, g-C3N4 hydrogel treated by 1 M sodium hydroxide, g-C3N4 hydrogel treated by 3 M sodium hydroxide, g-C3N4 hydrogel treated by 5 M sodium hydroxide, and g-C3N4 hydrogel treated by 8 M sodium hydroxide. Figure S2. The digital photo of g-C3N4 aerogel treated with different concentrations of sodium hydroxide corresponding to Figure S1. As can be seen, the g-C3N4 aerogel treated by 3 M and 5 M sodium hydroxide can hold well 3D structure, while the other two showed power-like structure. Figure S3. (a) TEM image of pristine g-C3N4; TEM image of GCNW after treatment with different concentrations of sodium hydroxide (b: 1 M, c: 3 M, d: 5 M). Figure S4. (a) SEM image of PEDOT: PSS. The illustration in the upper right corner is the photograph of PEDOT: PSS. (b, c) TEM images of 20% GCNW. The extracted elemental mapping images of C, N, O, and S, which indicate the homogeneous distribution of g-C3N4 nanowires and PEDOT: PSS. Figure S5. N2 sorption isotherms of g-C3N4 (a), GCNW (b), 50% GCNW/PEDOT: PSS (c), 20% GCNW/PEDOT: PSS (d). Figure S6. (a) S2 s XPS spectra of 20% GCNW. (b) Raman spectra of different mixing ratios of GCNW and PEDOT: PSS. In Figure S6b, two strong absorption peaks in the 1434 cm−1 and 1515 cm−1 region correspond to the symmetry Cα = Cβ (−O) stretching mode and the asymmetric C=C stretching mode which are characteristic of PEDOT: PSS. Figure S7. Electrochemical properties of pure PEDOT: PSS: (a) CV curve and (b) GCD curve. Figure S8. Electrochemical properties of 10% GCNW: (a) CV curve and (b) GCD curve. Figure S9. Electrochemical properties of 50% GCNW: (a) CV curve and (b) GCD curve. Figure S10. Electrochemical properties of 80% GCNW: (a) CV curve and (b) GCD curve. Figure S11. CV curves of the flexible device after 2000 bending cycles with 90°. Table S1. Summary of the capacitive performance of the supercapacitor [file 11671_2019_2932_MOESM1_ESM.docx]

Supporting Information

Three dimensional carbon nitride nanowire scaffold for flexible supercapacitors

Zhiwei Tang^1^, Xueyu Zhang^*2,3^, Lianfeng Duan^2^, Aimin Wu^3^, and Wei Lü^*2^

^1^School of Chemistry and Life Science, Changchun University of Technology, Changchun 130012, China

^2^Key Laboratory of Advanced Structural Materials, Ministry of Education & Advanced Institute of Materials Science, Changchun University of Technology, Changchun 130012, China

^3^Key Laboratory of Materials Modification by Laser, Ion, and Electron Beams (Ministry of Education), Dalian University of Technology, Dalian 116024, China

^*^E-mail: [dlut417@163.com](mailto:dlut417@163.com), lw771119@hotmail.com


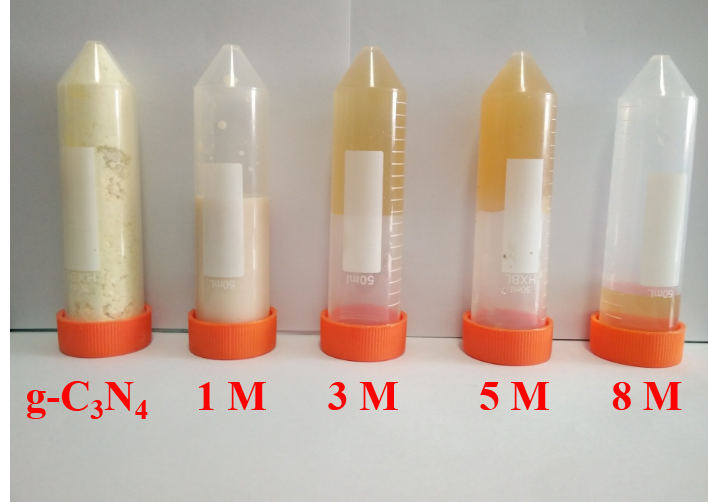


**Figure S1.** the digital photo of g-C_3_N_4_ hydrogel treated with different concentrations of sodium hydroxide. From left to right: pure g-C3N4 powder, g-C_3_N_4_ hydrogel treated by 1M sodium hydroxide, g-C_3_N_4_ hydrogel treated by 3M sodium hydroxide, g-C_3_N_4_ hydrogel treated by 5M sodium hydroxide, g-C_3_N_4_ hydrogel treated by 8M sodium hydroxide.


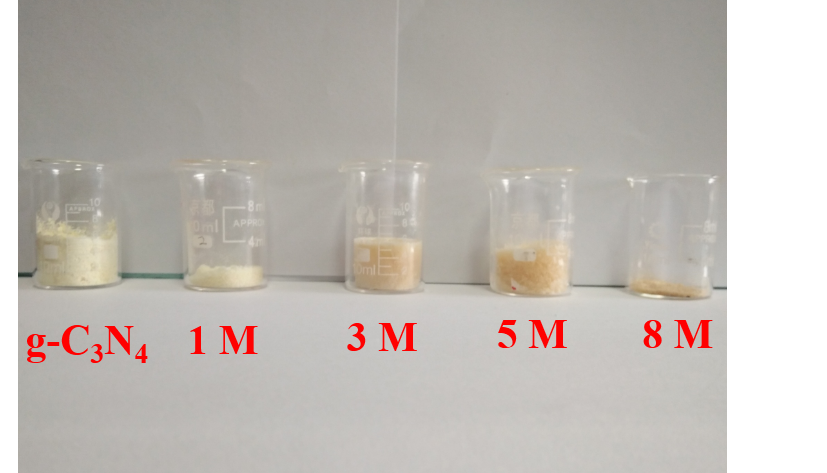


**Figure S2.** the digital photo of g-C_3_N_4_ aerogel treated with different concentrations of sodium hydroxide corresponding to Figure S1. As can be seen, the g-C_3_N_4_ aerogel treated by 3M and 5M sodium hydroxide can hold well 3D structure, while the other two showed power like structure.

***
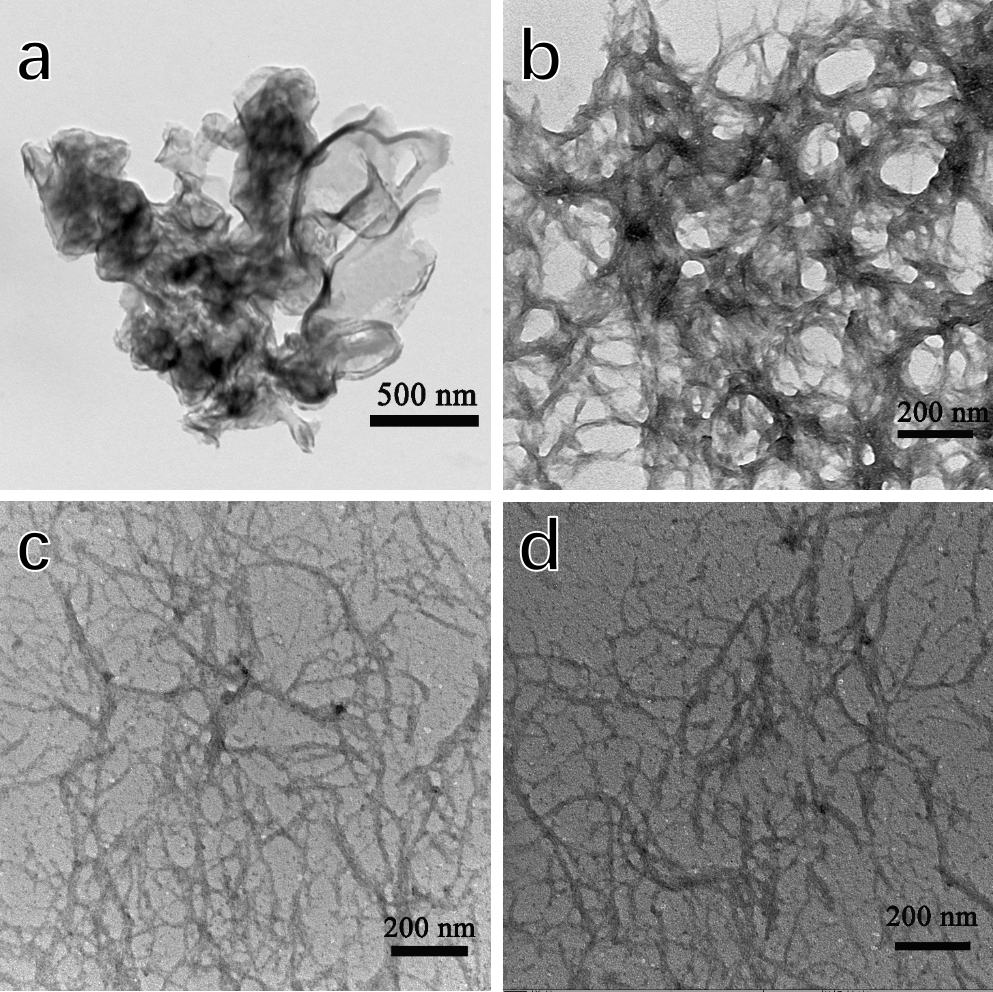
***

**Figure S3.** (a) TEM image of pristine g-C_3_N_4_; TEM image of GCNW after treatment with different concentrations of sodium hydroxide (b: 1M, c: 3M, d: 5M).

**
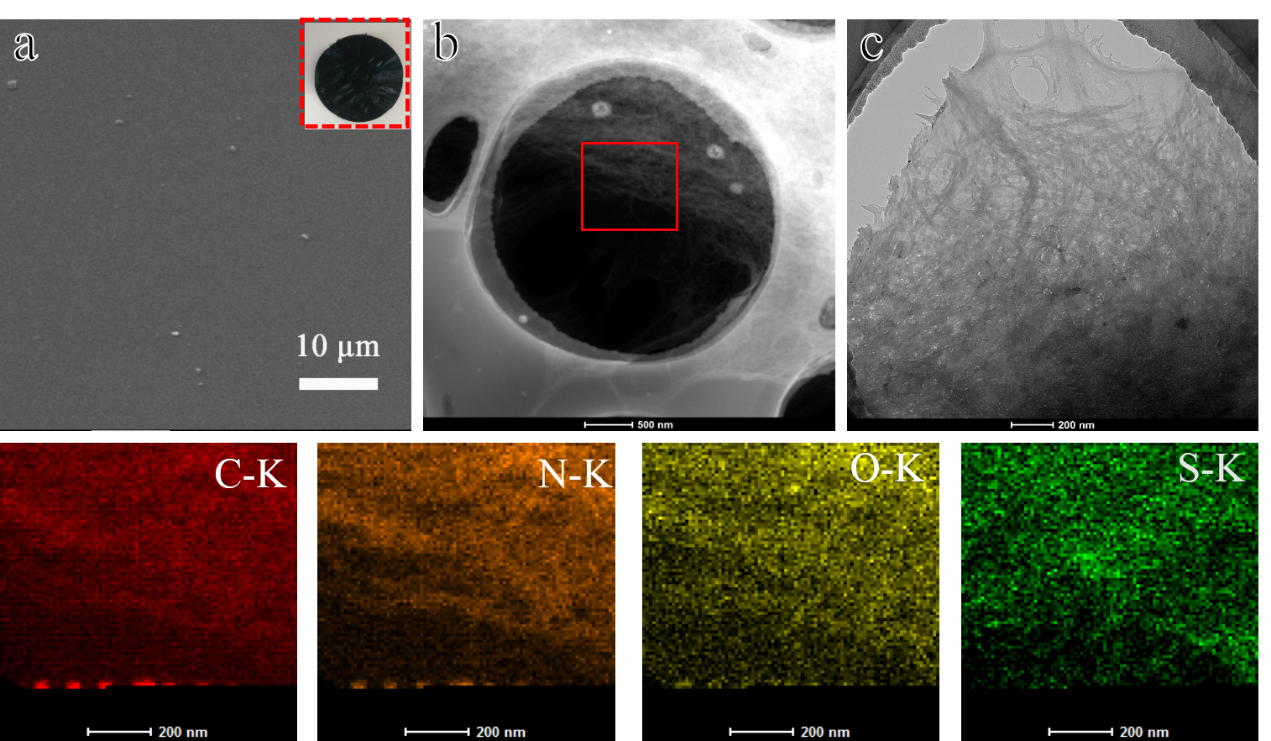
**

**Figure S4.** (a) SEM image of PEDOT: PSS. The illustration in the upper right corner is Photograph of PEDOT: PSS. (b, c) TEM images of 20% GCNW. The Extracted elemental mapping images of C, N, O and S, which indicate the homogeneously distribution of g-C_3_N_4_ nanowires and PEDOT: PSS.

**
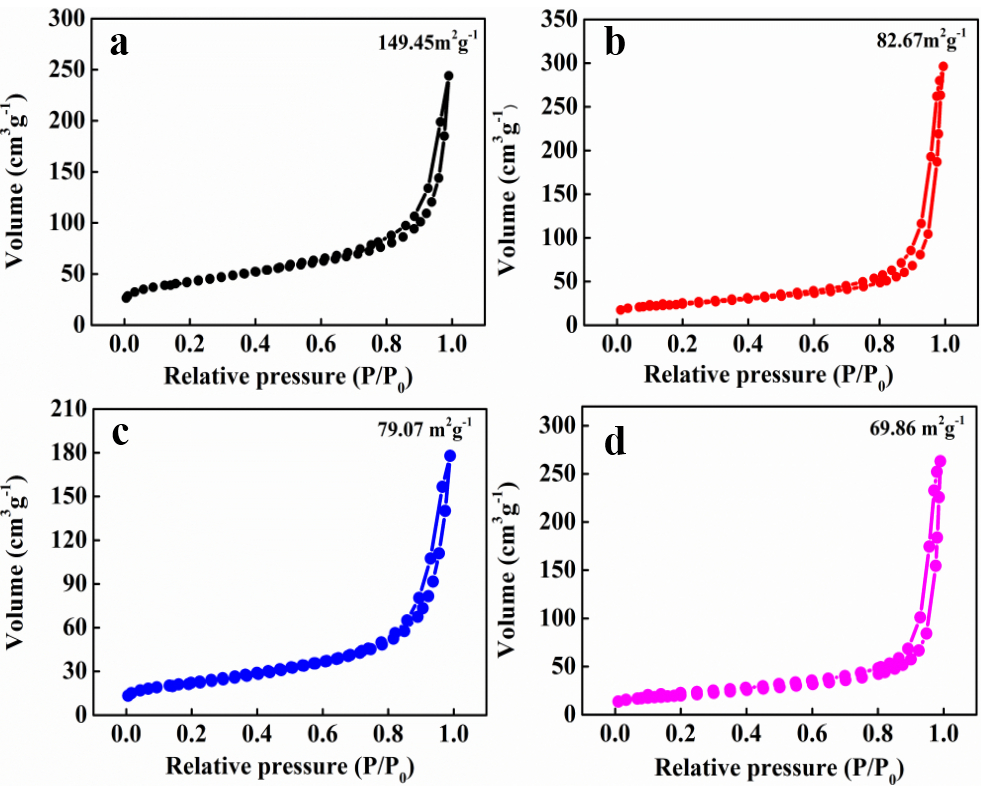
**

**Figure S5.** N_2_ sorption isotherms of g-C_3_N_4_ (a), GCNW (b), 50% GCNW/PEDOT: PSS (c), 20% GCNW/PEDOT: PSS (d).


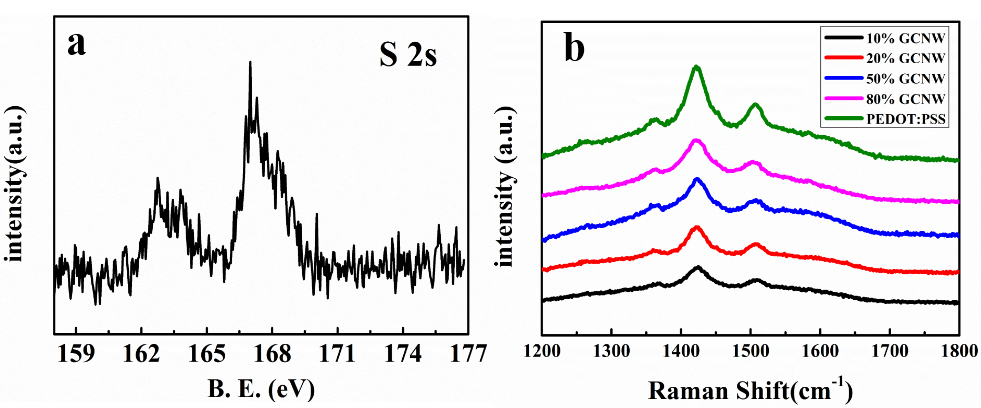


**Figure S6.** (a) S2s XPS spectra of 20% GCNW. (b) Raman spectra of different mixing ratios of GCNW and PEDOT: PSS. In Figure S6 b, two strong absorption peaks in the 1434 cm^-1^ and 1515 cm^-1^ region corresponds to the symmetry C_α_=C_β_ (-O) stretching mode and the asymmetric C=C stretching mode which are characteristic of PEDOT: PSS.

**
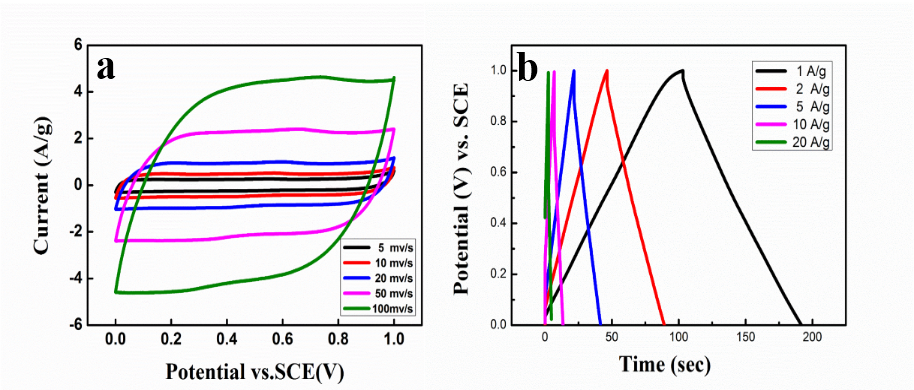
**

**Figure S7.** Electrochemical properties of pure PEDOT: PSS: (a) CV curve (b) GCD curve.


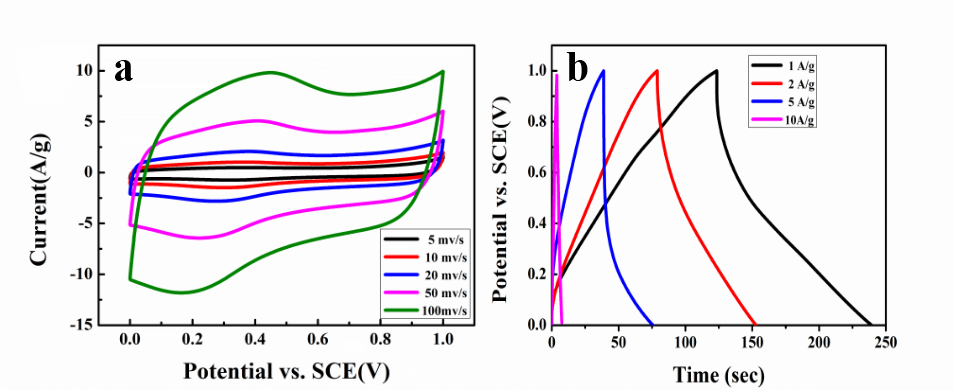


**Figure S8.** Electrochemical properties of 10% GCNW: (a) CV curve (b) GCD curve.


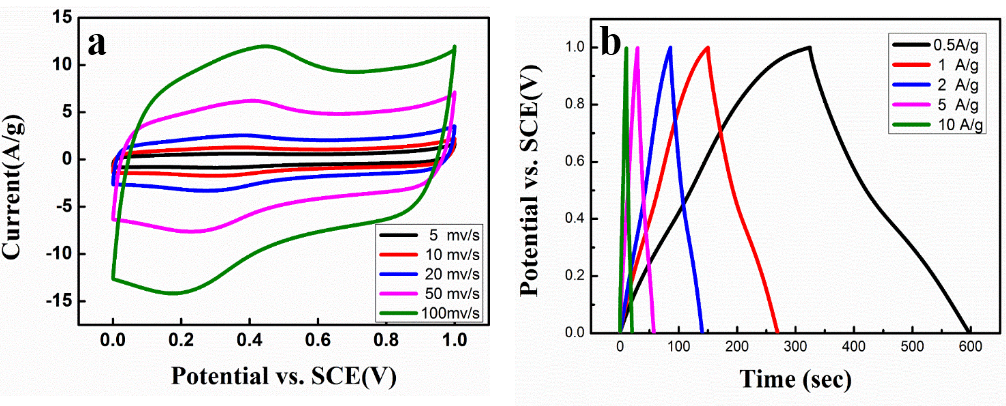


**Figure S9.** Electrochemical properties of 50% GCNW: (a) CV curve (b) GCD curve.


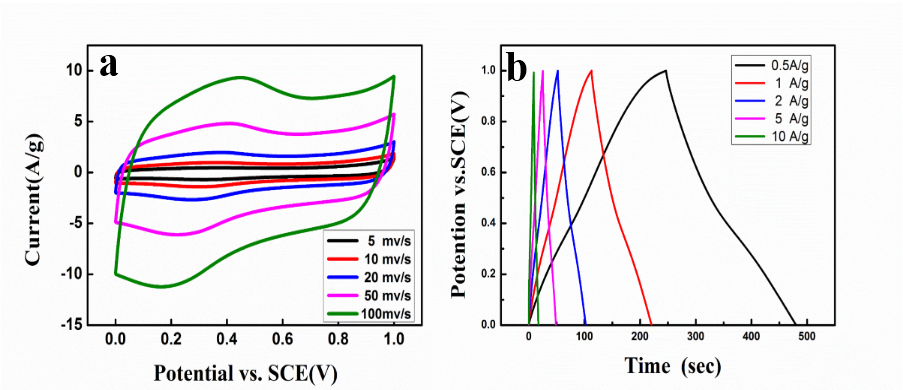


**Figure S10.** Electrochemical properties of 80% GCNW: (a) CV curve (b) GCD curve.


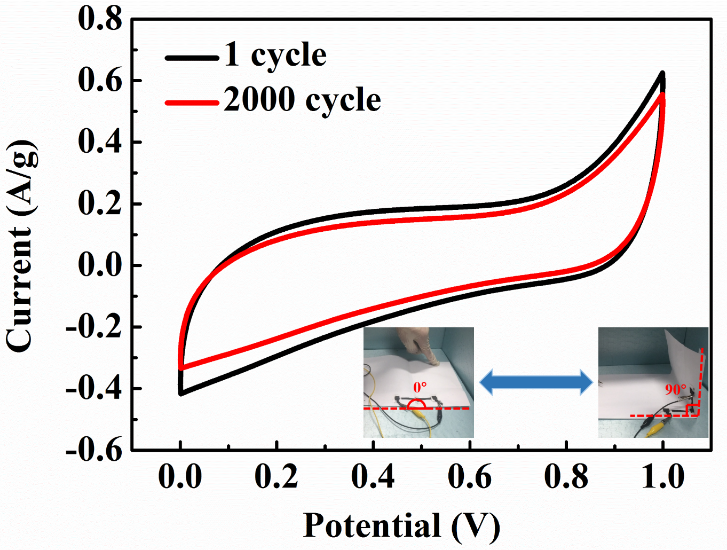


**Figure S11.** CV curves of the flexible device after 2000 bending cycles with 90°.

**Table S1** Summary of the capacitive performance of the supercapacitors based on similar structures

| **Materials** | **Electrolyte** | **Cs (F g^-1^)** | **Potential**  **Window (V)** | **R (Ω)** | **Ref.** |
| --- | --- | --- | --- | --- | --- |
| PEDOT | TBAPF_6_ | 70 | 1.0 | - | [36] |
| PEDOT:PSS/  CNT film | H_3_PO_4_ | 85.3 | 0.8 | 24.7 | [37] |
| PEDOT/  cellulose paper | H_2_SO_4_ | 145 | 1.2 | 3 | [38] |
| PEDOT:PSS/  Aramid Nanofibers | H_2_SO_4_ | 115 | 1.0 | 7.3 | [39] |
| PEDOT/  3D carbonnanotubes | H_2_SO_4_ | 147 | 1.0 | - | [40] |
| PEDOT-CNTs/GO | KCl | 91.6 | 1.0 | - | [41] |
| PEDOT nanopaper | H_2_SO_4_ | 128 | 1.0 | 1.7 | [42] |
| PEDOT:PSS/  cellulose nanofibrils  PEDOT:PSS/  Graphene films  Graphene/  Polypyrrole nanotube | HClO_4_  H_3_PO_4_  H_2_SO_4_ | 230  81  253 | 0.8    1.0    1.0 | ~10  -  0.36 | [43]  [44]  [45] |
| PEDOT:PSS/  GCNW | H_2_SO_4_ | 202 | 1.0 | 5.4 | This work |

Abbreviations: Cs, specific capacitance; CNT, carbon nanotube; R, internal resistance obtained from the electrochemical impedance spectra measurements.
